# Surgical leadership in Poland: ideas and challenges

**DOI:** 10.1515/iss-2019-0003

**Published:** 2019-05-04

**Authors:** Grzegorz Wallner, Michał Solecki

**Affiliations:** 2nd Department of General, Gastrointestinal and Oncological Surgery of the Alimentary Tract, Medical University of Lublin, Lublin, Poland

**Keywords:** education, leadership, national strategy, nontechnical skills, residency program, surgery

## Abstract

The Polish system of undergraduate and postgraduate medical education, including specialization courses in surgery, provided only general guidelines concerning the issue of creating a leader or preparing for leadership. The process of building the position of a leader has had a rather spontaneous character thus far; it has been based on the individual, natural predispositions of a candidate for the position of a leader. There are no formal guidelines for this in Poland. It is required that graduates of medical studies or residents should acquire the so-called professional and social skills before they complete their specialization training. In the light of the ongoing debate, it seems worthwhile to give a thought on the role of a leader and to undertake harmonized actions to work out a common stance on understanding the issue of leadership and teach leadership skills as a part of a harmonized, methodologically correct system of education, so that the best ways of preparing residents to perform the role of a leader in surgical and other medical surroundings could be realized.

In Poland, there are no formal guidelines or concrete theoretical assumptions for understanding the role of a leader or leadership in surgery. The Polish system of undergraduate and postgraduate medical education, including specialization courses in surgery, provided only general guidelines concerning the issue of creating a leader or preparing for leadership. The process of building the position of a leader has had a rather spontaneous character thus far; it has been based on the individual, natural predispositions of a candidate for the position of a leader.

What the curricula or programs of postgraduate courses pay special attention to are not only a proper level of theoretical knowledge but also individual practical skills. It is required that graduates of medical studies or residents should acquire the so-called professional and social skills before they complete their specialization training [1].

To get professional skills, the specialization training in surgery aims for residents to gain such a level of knowledge that will help them understand the basics of surgical treatment necessary for qualifying patients for operative treatment and for preoperative patient preparations as well as for performing surgical procedures independently. Following the guidelines included in the specialization training program, a surgeon should acquire necessary skills to be competent in treating emergency patients and arranged admissions and provide a full range of ambulatory surgical treatment and postoperative patient care; the doctors should also have optimal skills to assess the results of surgical treatment and its choice. Apart from individual specialty practice or providing health services in general surgery as a member of a medical team, a resident should also be trained in managerial skills so that he/she knows how to run a clinic, hospital ward, or outpatient surgical clinic; he/she should have appropriate skills to conduct specialization and in-service training in general surgery for other doctors or residents. It is assumed that the required professional competence also includes individual and teamwork organization skills and harmonized cooperation in interdisciplinary teams (consisting of different medical specialists, e.g. surgeons and anesthesiologists) as well as in interprofessional teams (a team of medical doctors, nursing staff, scrub nurses, medical rescuers, medical technicians, etc.). Unfortunately, up to the present day, the Polish educational system has not proposed concrete solutions to the problem of training residents to be competent in managing a surgical team [1], [2].

Bearing in mind the ongoing worldwide debate on this issue, it seems that to practice surgery it is important that, besides the skills of individual surgeons in broadening and updating their knowledge and gaining practical experience, they should also acquire the skills of a leader in everyday clinical practice and be able to be in charge of teamwork [3], [4], [5], [6], [7], [8]. This issue is of special importance at the stage of both diagnostic management and therapeutic management in the surgical unit, especially in the operating theater. It is well known that contemporary medicine is a discipline that requires teamwork [6], [8]. In these circumstances, the rules, which indicate the role of a leader in the team of operators or even of the whole therapeutic team, should be clearly determined and applied. This will guarantee a therapeutic success and obtain optimal surgical treatment results. According to the majority of scientific societies’ guidelines or experts’ recommendations, it is the surgeon who, quite naturally, will hold the position of a leader in the clinical practice requiring surgical care [6], [8], [9].

It seems understandable that acquiring the skills necessary to hold the position of the leader has become one of the priorities in the program of specialization training for some time now. Undoubtedly, it is the key to success in surgery [1], [5], [9]. All this is of the same importance as the hands-on practical training or gaining sufficient theoretical knowledge and clinical experience. The worldwide trends underline that it is a vital problem in the training of surgeons; therefore, it should be emphasized in all medical specialties independently of the level of training, rank, or clinical experience [3], [5], [6], [8], [9].

The operating suite, together with the operating theater with ongoing surgical procedures, is a special environment where a hierarchy of duties and work obligations should be observed and the role of a leader, coordinating all of the teamwork, is naturally assumed and held by the operator [4], [7]. Working in the operating theater creates a special, usually more stressful, microenvironment that demands focus and attention; the workplace is usually noisier than in other hospital units or outpatients’ clinics. Certainly, achieving a satisfactory level of coping in these conditions will require a lot of effort from the leader-to-be resident as well as his/her full commitment and methodologically correct training by experienced educators [3], [4], [5], [10].

The professional competence that each resident should acquire is expected to follow the recommendations of the European Union of Medical Specialists (UEMS: Union Européenne des Médecins Spécialistes) [11]. The aim of the basic module is to acquire sufficient knowledge and understand the basics of surgical treatment as well as to teach residents practical skills so that they are able to perform basic emergency procedures in the hospital and ambulatory treatment, so-called minor surgery, and provide basic management to postoperative patients [1], [10]. At present, there is an ongoing discussion on the necessity for residents to complete an obligatory training in basic social skills so that their awareness of personal qualities indispensible in the medical practice could be raised and certain features of character could be developed.

Social skills basically correspond to the English term “nonsurgical skills”. This type of competence also encompasses such skills as decision-making and readiness to take responsibility for one’s actions when working as a team. The same category of social competence includes organizational skills, i.e. the skills needed by the doctor in his/her own work as well as those necessary for harmonized cooperation within interdisciplinary and interprofessional teams [10]. Since the beginning of 21st century, the diagnostic-therapeutic decision-making relying on evidence-based medicine (EBM) as well as on the patient’s preferences has been put on the list of the social skills that need to be included in the training.

The aim of the general surgery module as part of the specialization training is to acquire knowledge and learn practical skills (both technical and nontechnical) necessary to perform surgical procedures on one’s own or accomplish clinical tasks independently [1], [10]. The guidelines for the specialization training with regard to surgical skills in the specialization module include an extended range of practical skills in comparison to the basic training module. Apart from learning to practice specialist medicine individually or provide services in general surgery as a member of a medical practice group, this training program assumes that the social skills competence should be extended and comprise the clinic, hospital ward, or general surgery outpatient clinic management as well as the ability to conduct a specialist training in general surgery for other doctors, in-service training of other medical personnel, and, finally, a training in supervising a medical experiment in general surgery that is in compliance with good clinical practice and EBM. With regard to the social skills, a special emphasis is put on the ability of decision-making and readiness to take responsibility for one’s own actions as well as for the team putting their trust in the leader; the ability to organize one’s own work and a harmonized cooperation within the team are also stressed. Having completed the specialization training module and confirmed a satisfactory level of acquisition of practical surgical and social skills, the doctor gets a diploma that certifies that he/she has gained qualifications necessary to manage a surgical unit independently [1].

Unfortunately, apart from general recommendations, the program of specialization training in general surgery in Poland does not provide concrete solutions or methodologically correct forms or methods of teaching leadership in surgery. By and large, this issue is expected to be decided by the doctor in charge or the resident’s coach. To some extent, it depends on one’s own experience, ingenuity, or knowledge. As a rule, every surgical ward or specialization manager works out his/her own way of teaching leadership in surgery based on the local training traditions, the region they practice in, and the historical roots of a particular medical school. In the Polish system of specialization training in surgery, we definitely follow the patterns and traditions of German surgery.

In the recent years, because of more frequent visits of Polish surgeons to the western countries and more frequent attempts to carry out the specialization training following the western, Anglo-Saxon methods, both British and primarily American, and remain in compliance with the UEMS recommendations and obligatory paradigm of surgery practice, including EBM, evidence-based surgery, and evidence-based education, the need for a methodologically correct leadership in all surgical specialties training has been gaining recognition in Poland. Appropriate courses and workshops are organized to serve this purpose, but they focus more on general professional career matters than on the surgeons’ issues. At present, a heated debate on this issue is going on among experts representing different surgical disciplines. The National Consultants and the Association of Polish Surgeons (APS) together with the Ministry of Health have made a decision to form three to five centers of simulation training in Poland, in which by definition, except practical training, the leadership in surgery training will be conducted, too. The Polish National Consultant in Surgery (PNCS) is responsible for initiating national epidemiological studies in the field of general surgery including the evaluation of study results. Additionally, the PNCS is the main advisor for the National Health Program in the field of surgery and other health programs approved by the Ministry of Health. The PNCS prognoses and creates the map of health needs of the Polish population. Moreover, the PNCS provides pieces of advice to the National Centre of Medical Exams and the Centre of Postgraduate Medical Education. The plan of changes in specialization training has been named Surg Excellence [10]. The changes included a systematic incorporation of simulation-based methodology to four core courses: fundamentals of laparoscopic surgery, ultrasonography in surgery, endoscopic procedures in general surgery, and training in operating room including perioperative care. One of the core points was to train surgical medical educators that elaborated a detailed training program. Cadaveric training and training on animals are supposed to be incorporated. Additionally, the new program includes elements of leadership in surgery, teamwork, and crisis resource management (CRM), especially in the operating room. As some of this was not incorporated in a systemic way to the curricula, a great shift and redefinition of the role of a surgeon as a periodical and potential leader but especially team player is expected. Apart from the traditional localization of surgical residency, i.e. in clinical surroundings, and covering the simulation module, the program encompasses elements of residents’ training first on cadavers and next on animals as well as teaching them how to conduct scientific research on surgery. In this matter, an agreement has been reached between the National Consultants and scientific societies representing all surgical disciplines. The developed concept of changes in surgical training in Poland accepts the principles recommended by the UEMS, with its Section of Surgery & European Board of Surgery that embraces the surgery representatives from the European Union (EU) countries, as well as the standards of the American College of Surgeons or the American Board of Surgery, a nongovernmental, nonprofit organization representing mainly surgical organizations in the United States and a member of the American Board of Medical Specialties [8], [11].

Moreover, the conception of teaching the leadership/medical team management skills at the beginning of medical education as a pre-diploma training has also been accepted. As a signatory state of the Bologna Declaration, we are obliged to respect the agreements of this Declaration. Understanding the need for introducing major changes in this aspect of training has resulted in the radical reorganization of the medical education system. For three years now, all the medical universities in Poland have been developing a network of university centers of medical simulation for training medical professionals and, among other things, to prepare both undergraduates and graduates of medical schools for leadership.

Intensive recruitment and training of certified educationists are also in place. The Medical University of Lublin, the workplace of the authors of this paper, has the leading position in Poland. For a few years to now, the Medical University of Lublin has been training educators and gradually introducing such classes in surgery for medical students, which are in agreement with the current trends and are also methodologically correct, including the leadership training that aims at acquisition of the leadership skills necessary to fulfill individual therapeutic tasks as well as teamwork.

The operating theater is a place where the leadership skills are especially significant for surgeons. Basic rules that establish or characterize the position of a leader in surgery have been identified. Since 2018, this purpose has been fulfilled by the realization of the SafEAST grant “Safety and Interdisciplinary Surgical Care” financed by the Ministry of Health from the EU funds. Educators, general surgeons, and a psychologist from the Centre of Medical Simulation at the University of Lublin designed the program. The Medical University of Lublin’s Simulation Centre, in fact, is a copy of a hospital that attending students named “Hospital of the Future”. Within the facility, the emergency department, hospital wards, pharmacy, operating rooms, and intensive care unit were placed in combination with a self-developed software, which created a very realistic hospital environment. The concept of the SafEAST program was to develop a training curriculum where residents at early stages of education (first 2 years) from the field of general surgery get acquainted with a perioperative environment in an interprofessional, interdisciplinary, and safe environment. In cooperation with a psychologist and a specialist in medical simulation and medical communication, decision trees and high-fidelity scenarios were created. One of the principal goals of the project was to incorporate the CRM-8 goals (Know your environment; Anticipate, share, and review the plan; Provide effective leadership; Ensure role clarity and good teamwork, Communicate effectively; Call for help early; Allocate attention wisely – avoid fixation; and Distribute the workload – monitor and support team members) into the clinical practice of young residents based on a simulation environment. Teachers involved in the training were general surgeons. As the project required a participation of simulated patients, an additional trainer was involved for this role. The first stage of the plan was designed to develop the leadership skills independently of the represented discipline and comprised the identification of the residents’ strong and weak sides. In each resident, the competence of a potential leader is assessed in two aspects: with reference to the individual features of a leader and with reference to the qualities predisposing the resident to become a leader and be in charge of a team. One of the elements of a surgeon’s evaluation is the so-called emotional intelligence and whether he/she has abilities enabling him or her to control and understand his/her own emotions as well as the emotions of the members of the operating team. All this allows the residents in training to acquire correct and constructive interpersonal communication skills that guarantee optimal diagnostic-therapeutic decision-making. The SafEAST program has been scheduled between 2019 and 2021. The first editions took place in March 2019.

As mentioned previously, in Poland, it is mainly achieved by the implementation/adaptation of the earlier experience gained by a surgical center rather than by a methodologically hammered out model of teaching the leadership skills in surgery. Depending on the tradition in individual surgical wards in Poland, different styles of practicing surgery, including specialization training in surgery, have been identified. That is why several different categories or styles of leadership in surgery may be observed in the work of Polish surgical wards. Following the authoritarian style, the leader presents the aims to his/her coworkers in the long-term perspective and makes efforts to give them support while realizing the planned goal. A leader or a mentor or a coach type helps the team members to identify their weak and strong sides (features), thereby motivating them to look for their own solutions that will optimize their professionalism. Creating a friendly working environment for coworkers, which helps in building a good rapport, trust, and loyalty as well as in establishing high moral standards, is another form of leadership. A democratic leader, on the contrary, is open to new ideas or solutions suggested by the surgical team members and tries to obtain optimal results collectively. Still another form of leadership relies on determining high working standards at the start and mobilizing the team to raise the bar in terms of the surgeon’s work quality and efficacy. Lastly, there are surgical wards where the leaders, like a commander in chief in the military forces, give orders and demand their immediate execution.

In any of the above presented category, the interdisciplinary and interprofessional communication skills are of utmost importance. They are absolutely essential in surgical emergencies/crises that are commonplace in the surgical practice, especially in the operating theater. More often than not, it is the communication among the members of the surgical team, operating block members, and members of the occupational group that guarantees the ultimate therapeutic success. Unfortunately, in Poland, the proper evaluation of surgical leadership lacks both monitoring and methodologically correct analysis of the quality improvement in the current systemic surgical care.

Apart from the above-analyzed leadership in the surgical suite surroundings or leadership in a surgical unit, there is one more aspect of this issue labeled as leadership in surgery in Poland. It can be seen in a broader sense as follows: How is the leadership understood in the Polish surgery? Certainly, by definition, leadership encompasses all the activities or abilities to accomplish tasks individually, i.e. by a single person who is called a leader, or by a group of individuals (leaders) who are the CEO or the managing director in a corporation or the president of a scientific society representing a certain professional group or community; in our case, it is a representation of the community of surgeons. Therefore, an individual surgeon or an organization embracing surgeons in Poland who, to meet the leadership in surgery criteria, should have a certain vision that will give a vital perspective and thereby set goals/directions to be followed by the whole surgical community.

From my understanding, a leader in surgery should also be a moral authority, highly professional in the specialty or discipline he/she represents, and a role model for other surgeons to look up to.

A leader should be a progress initiator, determines the rules, and mobilizes the members of his/her community to increase professional activity/efficacy. A leader is expected to dominate and have distinctive features that make him or her stand out from the whole group. A leader must inspire others, affect them positively, have the ability to convince them to their own decisions, and motivate further development. It is difficult to give one characteristic of a leader or leadership. To a certain extent, it is determined by the community and connected with individual personalities.

However, there are features that individual or group leaders need to have in common. The features characteristic of an individual leader or a leadership group include determination and a thorough knowledge of the conditions and circumstances in which the community functions. The leader or leaders should also be able focus on the essential, most important problems of the community. A leader should be a good negotiator and be effective in achieving and building consensus through participation in all activities advantageous to the community.

Moreover, a leader should also broaden his/her mind, perfect his/her skills and expect the members of his/her team to follow in his/her footsteps. In contrast, a leader should not focus on satisfying his/her own needs but put the interest of surgical community his/her represent first. Yet, leadership is not about managing people or “playing people” but rather about initiating activities and building a rapport based on mutual trust, and all this will result in improved efficacy and increased independence of the members of a particular community. A leader should definitely be identified by his/her own success but also by each individual success of all his/her community members. The managerial system in medical specialties, including surgery, in Poland is based on a certain centralization of activities at the national level and at the level of individual regions. While analyzing the issue of individual leadership or joint leadership in Poland, three or even four levels of surgical community activities emerge. First of all, there is the Medical Council with the President of the Medical Association as its Head with a section designed to see the Polish surgeons’ issues. With the authorization of the Health Secretary and with an approval of the whole surgical community embraced by the Polish Medical Association as well as by the recommendation of the APS, the specialist supervision in surgery is exercised by the National Consultant and Regional Consultants in surgery. Lastly, in the surgical community in Poland, various scientific societies are engaged and their leading representative is the APS with its President, Board, and APS regional branches. Certainly, the APS has different active sections with the democratically elected society or section authorities.

There may also be naturally created individual or group leaders setting the tone for activity in a particular group of surgeons representing a specific specialty or discipline of treatment and who come across as professionally active, e.g. recognized clinical experts, accomplished specialists in surgery or those with considerable scientific achievements, and individuals standing out because their activities are advantageous for the surgical community in Poland.

If a surgeon becomes involved in the activities of the Medical Council, he/she will deal definitely with administrative-social, legal, and union issues. It seems, however, that when it comes to qualifying for leadership it is more about one’s real, professional interaction with the members of the surgical community and about a stand-out personality, an acknowledged clinician in surgery, or a surgeon with exceptional scientific achievements and respected by the surgical community.

To some extent, these criteria, at least theoretically, are met by National Consultants in surgery and also by the President of the APS. These two positions must be objectively verified in the democratic elections, which, as a rule, gives grounds for an official mandate to assume the position of a leader and at the same time provides a substantive argumentation for their becoming a natural leader in the surgical community. The choice of these candidates is certainly preceded by many years of their professional activity, building up moral authority, involvement in scientific society matters, and work for the benefit of the surgical community in Poland.

Because of the held positions, the National Consultants or the President of the APS have a range of tasks and duties that have to be in the agreement with legal regulations or with the statute of the APS. In each case, however, it is not about executing duties and the realization of tasks formally but rather about an individual approach to the management or even undertaking disinterested activities advantageous for the surgical community.

In the light of the ongoing debate, it seems worthwhile to give a thought on the role of a leader and to undertake harmonized actions to work out a common stance on understanding the issue of leadership and teach leadership skills as a part of a harmonized, methodologically correct system of education, so that the best ways of preparing residents to perform the role of a leader in surgical and other medical surroundings could be realized.

## Supplementary Material

The article (iss-2019-0003) offers reviewer assessments as supplementary material.
