# Supplementary material for: Surgical leadership in Poland: ideas and challenges
Source: Innov Surg Sci. 2019 May 4;4(2):45–50. doi: 10.1515/iss-2019-0003 (PMC6754058; doi:10.1515/iss-2019-0003)
Supplement: Supplementary file 1 [file iss-4-20190003-s001.pdf]

Reviewer Assessment

Grzegorz Wallner* and Michał Solecki

Surgical leadership in Poland: ideas and challenges

https://doi.org/10.1515/iss-2019-0003
Received March 7, 2019; accepted April 8, 2019

***Corresponding author: Grzegorz Wallner,** 2nd Department of General, Gastrointestinal and Oncological Surgery of the Alimentary Tract, Medical University of Lublin, Lublin, Poland, E-mail: gtwallner@gmail.com

Reviewers' Comments to Original Submission

Reviewer 1: Matthias Rothmund

Mar 14, 2019

**Reviewer Recommendation Term:**	Accept with Minor Revision
**Overall Reviewer Manuscript Rating:**	90
**Custom Review Questions**	**Response**
Is the subject area appropriate for you?	5 - High/Yes
Does the title clearly reflect the paper's content?	4
Does the abstract clearly reflect the paper's content?	5 - High/Yes
Do the keywords clearly reflect the paper's content?	4
Does the introduction present the problem clearly?	4
Are the results/conclusions justified?	4
How comprehensive and up-to-date is the subject matter presented?	4
How adequate is the data presentation?	N/A
Are units and terminology used correctly?	N/A
Is the number of cases adequate?	N/A
Are the experimental methods/clinical studies adequate?	N/A
Is the length appropriate in relation to the content?	3
Does the reader get new insights from the article?	4
Please rate the practical significance.	4
Please rate the accuracy of methods.	N/A
Please rate the statistical evaluation and quality control.	N/A
Please rate the appropriateness of the figures and tables.	N/A
Please rate the appropriateness of the references.	3
Please evaluate the writing style and use of language.	3
Please judge the overall scientific quality of the manuscript.	4
Are you willing to review the revision of this manuscript?	Yes

Comments to Authors:

Open Access. © 2019 Wallner G., Solecki M., published by De Gruyter. CC BY This work is licensed under the Creative Commons Attribution 4.0 Public License.

This author is to be congratulated to adress an important issue in surgical education, i.e. the importance of adding non-surgical skills to the standard training of young surgeons. He emphasizes a new approach in Polish medical and surgical education in this regard, especially in 5 academic centers.
It has to be mentioned that Poland is not the only country where non-surgical skills are not taught during formal surgical training. This is true for almost all countries.
To the reviewers knowledge the Royal College of Physicians and Surgeons in Canada was the first to describe a modern framework of competencies of future physicians and surgeons in 2000. (Frank, J.R., Langer, B., World J. Surg. 27, 972-978, 2003). According to this CanMEDS project, medical specialists should be: Experts in their specialty, Managers, Health Advocates, Scholars and Professionals and - this was new - Communicators and Collaborators. It was the first time that communication and social skills were identified to be important in medical/surgical education. Also it was clear that these virtues were not innate natural abilities but could be learned.
The author leads these thoughts further and describes recent developments in his country.

To improve the manuscript the reviewer proposes the following:

1. the author should focus more on the activities in the 5 Polish centers or in his own university and describe more in detail how non-surgical skills are taught. He especially should describe in what stage the SEFEAST project „Safety and interdisciplinary surgical care" is and also in detail what is or will be learned, who are the teachers and at what stage of their surgical education are the young doctors who attend.

2. instead the chapters „Introduction" and „Professional skills - knowledge or competency" can be shortened, e.g. the idea that each graduate or resident should acquire social skills is mentioned 3 times (page 2, 2nd paragraph, page 3,2nd and 3rd paragraph), also on page 4, 2nd paragraph of the chapter „Residency training in Poland" and on page 5, 5th line. Also it is repeatedly said that „ this competence encompasses decision-making and readiness to take responsibility(page 4, last p. and in the middle of page 5.

3. What does the author think of learning soft skills from role models, though they can be formally taught? Can he comment on this? If it comes to top leaders, learning from role models can be a good tool (Rothmund, M. Surgical leadership, Brit. J. Surg., 2013;100: 577–579)

4. On page 3, last p., the author claims that in a operating suite the surgeon has the final say. What is his opinion on a „suite organizer", who in Germany is rarely a surgeon?

5. When it comes to organisations, the author should explain what „National Consultant" or „Regional Consultants" mean.

6. Finally, the author should comment on forming not only leaders but also team players in the surgical world, where nowadays hierarchies are more often flat compared to the past.

I recommend to accept this manuscript with minor revisions.

Reviewer 2: anonymous

Mar 14, 2019

**Reviewer Recommendation Term:**	Accept with minor revision
**Overall Reviewer Manuscript Rating:**	67
**Custom Review Questions**	**Response**
**Comments to Authors:**	
Is the subject area appropriate for you?	5 - High/Yes
Does the title clearly reflect the paper's content?	5 - High/Yes
Does the abstract clearly reflect the paper's content?	5 - High/Yes
Do the keywords clearly reflect the paper's content?	5 - High/Yes
Does the introduction present the problem clearly?	4
Are the results/conclusions justified?	5 - High/Yes
How comprehensive and up-to-date is the subject matter presented?	5 - High/Yes
How adequate is the data presentation?	4

Are units and terminology used correctly?	4
Is the number of cases adequate?	N/A
Are the experimental methods/clinical studies adequate?	N/A
Is the length appropriate in relation to the content?	4
Does the reader get new insights from the article?	4
Please rate the practical significance.	5 - High/Yes
Please rate the accuracy of methods.	N/A
Please rate the statistical evaluation and quality control.	N/A
Please rate the appropriateness of the figures and tables.	N/A
Please rate the appropriateness of the references.	4
Please evaluate the writing style and use of language.	4
Please judge the overall scientific quality of the manuscript.	4
Are you willing to review the revision of this manuscript?	Yes

The author is describing ideas about the Polish system of undergraduate and postgraduate medical education and the challenges due to a lack of formal and binding guidelines concerning the issue of creating a leader or preparing for leadership. How the leadership in the Polish surgery is understood, depends on the tradition in individual surgical wards all over the country and different styles of practicing surgery, including specialist training in surgery.

The surgical community in Poland comprises various scientific societies. Their leading representative is the Association of Polish Surgeons (APS) with its President, Board and APS regional branches, responsible together with a not in more detail explained „National Consultant“ and the Ministry of Health for creating 3-5 centers of simulation training in Poland. There furthermore practical training the leadership in surgical training will be conducted too, with „SurgExcellence“ creating a new concept changing specialization training. That is in contrast to Germany, where the responsibility of postgraduate education and specialization is transferred from the government to the German Medical Association (Bundesärztekammer) with its federal state medical associations (Landesärztekammern). The scientific surgical societies only have consulting functions and competencies.

Undoubtedly residents should acquire adequate professional and social skills before they complete their specialization training. Surgery always has been, and always will be, a craft specialty. Therefore the acquisition of psychomotor skills is essential, but this must be done on the background foundation of adequate knowledge of other skills such as communication and clinical judgement. In mentoring programs of the past, all too often the teaching of operative surgery followed the age-old aphorism of ‚see one, do one, teach one‘. It has represented the model for surgical education for over a century, however recent changes in education have reduced autonomy in training. This is no longer acceptable, not only on the grounds of clinical governance and patient safety, but also on the basis of professionalism [1].

In today‘s more complex health care institutions, leadership, management and business skills matter equally. Specific programs should be developed in order to equip faculty members with these skills, thus allowing them to deliver a new surgical curriculum, teach surgical proficiency on courses and in the operating theatre, test competency and to play the role of a mentor.

In the UK, the four surgical Royal Colleges have joined together to develop an explicit surgical curriculum. This has been based on the CanMEDS roles, described in 2000 by the Royal College of Physicians and Surgeons of Canada as skills for the new millennium. According to this program a surgical expert should be defined as medical expert (in the integrative sense), communicator, team player, health advocate, manager, scholar and professional. Key competencies are assigned to these different roles, defining what a specialist must be able to do [2]. Considering training methods in surgery, it is important to recognize the principles of adult education. These principles were the basis for the Training the Trainers Manual: Learning and Teaching of the Royal College of Surgeons of England (RCSE) [3]. In cooperation with the RCSE the manual was transformed into a course by the German Society of Surgeons. Unfortunately this concept was only poorly received by the German Medical Association, which is responsible for the postgraduate medical education in Germany. The program was offered by the Surgical Society as an optional course, but was stopped due to a lack of uptake by residents in preparation for leading roles in surgery. Teach the teacher programs are offered from some medical faculties or university hospitals. The Harvard Surgical Leadership Program [4] for example is a postgraduate certificate program for surgeons seeking to step into and succeed in leadership positions and acquire the skills needed to excel as heads of departments, divisions, projects, and institutions at large. The curriculum highlights modern leadership strategies and executive skills for surgeons, the role of the surgeon as an entrepreneur and innovator and legal principles for surgical leaders.

Within Europe, the working time directive has led to a system of shifts and rotas, which has along with a shorter overall period of training led to reduced time available to surgical trainees in which to learn their craft. In the light of this reduced training time, assessment of surgical competency is becoming even more important and should be a professional priority as well with patient safety and political imperatives in mind. It remains a concern that extended shifts in medical residency programs may adversely affect patient safety but also regarding the effect of current surgical resident duty-hour policies on patient outcomes, resident education, and resident well-being. Allowing program directors flexibility in adjusting duty-hour schedules for trainees did not adversely affect 30-day mortality or several other measured outcomes of patient safety [5]. Compared with standard duty-hour schedules, flexible, less-restrictive duty-hour policies for surgical residents could not be associated with inferior patient outcomes and showed no significant difference in residents‘ satisfaction with overall well-being and education quality [6].

1. Thomas WEG. Teaching and Assessing Surgical Competence. Ann R Coll Surg Engl. 2006 September; 88(5): 429-432
2. Skills for the new millennium: The CanMeds 2000 Projekt chttp://www.royalcollege.ca/rcsite/canmeds/canmeds-framework-e (11.3.2019)
3. The Royal College of Surgeons of England. Training the Trainers Manual: Learning and Teaching. London: RCSE, 2006.
4. Harvard Surgical Leadership Program https://s3.amazonaws.com/hms-surgery/Harvard+Medical+School+-+Surgical+Leadership+Program.pdf (11.3.2019)
5. Silber JH, Bellini LM, Shea JA et al for the iCOMPARE Research Group. Patient Safety Outcomes under Flexible and Standard Resident Duty-Hour Rules. N Engl J Med 2019; 380:905-914
6. Bilimoria KY, Chung JW, Hedges LV et al. National Cluster-Randomized Trial of Duty-Hour Flexibility in Surgical Training. N Engl J Med 2016; 374:713-727

Surgical competence and its assessment is one of the most hotly debated topics engaging the profession. The present paper gives an overview and describes how leadership is understood and imparted to candidates for the position of a leader in Polish surgery. After the reorganization of the education system, the paper is based on three years of experience of the leading institution for intensive recruitment and training of certified educationists at the Medical University of Lublin, the workplace of the author of this paper.
In the ongoing debate about surgical leadership it seems a worthwhile contribution. The paper it is recommended for publication in the present form after a minor revision:
Page 6: Please explain the term and meaning of „National Consultant“ and the Concept of SurgExcellence
Page 7: Please give a short comment to the status of implementation of the SEFEAST project „Safety and interdisciplinary surgical care“

Authors' Response to Reviewer Comments

Reviewers' Comments to Revision

29 March 2019

Respected Editor and Reviewers,

Thank You very much for considering my manuscript into publication in your distinguished journal. Additionally thank you for taking your time for editing, reviewing and sharing your comments. After a detailed revision of the sent reviewers' and editor's comments I would like to reply to the suggested changes and questions.

Reviewer #1

This author is to be congratulated to address an important issue in surgical education, i.e. the importance of adding non-surgical skills to the standard training of young surgeons. He emphasizes a new approach in Polish medical and surgical education in this regard, especially in 5 academic centers.
It has to be mentioned that Poland is not the only country where non-surgical skills are not taught during formal surgical training. This is true for almost all countries.
To the reviewers knowledge the Royal College of Physicians and Surgeons in Canada was the first to describe a modern framework of competencies of future physicians and surgeons in 2000. (Frank, J.R., Langer,B., World J.Surg. 27,972-978, 2003). According to this CanMEDS project, medical specialists should be: Experts in their specialty, Managers, Health Advocates, Scholars and Professionals and - this was new - Communicators and Collaborators. It was the first time that communication and social skills were identified to be important in medical/surgical education. Also it was clear that these virtues were not innate natural abilities but could be learned.

Author's Reply (AR): Thank you for this valuable publication. It was cited and incorporated to the manuscript.

The author leads these thoughts further and describes recent developments in his country. To improve the manuscript the reviewer proposes the following:

1. the author should focus more on the activities in the 5 Polish centers or in his own university and describe more in detail how non-surgical skills are taught. He especially should describe in what stage the SEFEAST project „Safety and interdisciplinary surgical care" is and also in detail what is or will be learned, who are the teachers and at what stage of their surgical education are the young doctors who attend.

AR: The description of the SafEast Program including learning goals, preparation team and attendees was described at page No.7

2. instead the chapters „Introduction” and „Professional skills - knowledge or competency” can be shortened, e.g. the idea that each graduate or resident should acquire social skills is mentioned 3 times (page 2, 2nd paragraph, page 3,2nd and 3rd paragraph), also on page 4, 2nd paragraph of the chapter „Residency training in Poland” and on page 5,5th line. Also it is repeatedly said that „ this competence encompasses decision-making and readiness to take responsibility(page 4, last p. and in the middle of page 5.

AR: the paragraphs and repetitions were corrected or deleted.

3. What does the author think of learning soft skills from role models, though they can be formally taught? Can he comment on this? If it comes to top leaders, learning from role models can be a good tool (Rothmund,M. Surgical leadership, Brit.J.Surg., 2013;100: 577-579)

AR: I think that role models have a different utility and it’s not free from limitations. There is evidence suggesting the effectiveness in this method. Our experience (program in communication skills in surgery) for residents showed limited satisfaction of training participants (post training survey).

4. On page 3, last p., the author claims that in a operating suite the surgeon has the final say. What is his opinion on a „suite organizer”, who in Germany is rarely a surgeon?

AR: As the role of the surgeon is different in different locations among the Republic this sentence was deleted. Additionally was not precisely defined.

5. When it comes to organisations, the author should explain what „National Consultant” or „Regional Consultants” mean.

AR: The role of Polish National Consultant was explained in P.5-6 including the description of the SurgExcellence program P.6

6. Finally, the author should comment on forming not only leaders but also team players in the surgical world, where nowadays hierarchies are more often flat compared to the past.
AR: This remark was added to the Page 6

I recommend to accept this manuscript with minor revisions.

Reviewer #2: The author is describing ideas about the Polish system of undergraduate and postgraduate medical education and the challenges due to a lack of formal and binding guidelines concerning the issue of creating a leader or preparing for leadership. How the leadership in the Polish surgery is understood, depends on the tradition in individual surgical wards all over the country and different styles of practicing surgery, including specialist training in surgery.
The surgical community in Poland comprises various scientific societies. Their leading representative is the Association of Polish Surgeons (APS) with its President, Board and APS regional branches, responsible together with a not in more detail explained “National Consultant” and the Ministry of Health for creating 3-5 centers of simulation training in Poland. There furthermore practical training the leadership in surgical training will be conducted too, with “SurgExcellence” creating a new concept changing specialization training. That is in contrast to Germany, where the responsibility of postgraduate education and specialization is transferred from the government to the German Medical Association (Bundesärztekammer) with its federal state medical associations (Landesärztekammern). The scientific surgical societies only have consulting functions and competencies.
Undoubtedly residents should acquire adequate professional and social skills before they complete their specialization training. Surgery always has been, and always will be, a craft specialty. Therefore the acquisition of psychomotor skills is essential, but this must be done on the background foundation of adequate knowledge of other skills such as communication and clinical judgement. In mentoring programs of the past, all too often the teaching of operative surgery followed the age-old aphorism of ‘see one, do one, teach one’.
It has represented the model for surgical education for over a century, however recent changes in education have reduced autonomy in training. This is no longer acceptable, not only on the grounds of clinical governance and patient safety, but also on the basis of professionalism [1].

In today's more complex health care institutions, leadership, management and business skills matter equally. Specific programs should be developed in order to equip faculty members with these skills, thus allowing them to deliver a new surgical curriculum, teach surgical proficiency on courses and in the operating theatre, test competency and to play the role of a mentor.

In the UK, the four surgical Royal Colleges have joined together to develop an explicit surgical curriculum. This has been based on the CanMEDS roles, described in 2000 by the Royal College of Physicians and Surgeons of Canada as skills for the new millennium. According to this program a surgical expert should be defined as medical expert (in the integrative sense), communicator, team player, health advocate, manager, scholar and professional. Key competencies are assigned to these different roles, defining what a specialist must be able to do [2]. Considering training methods in surgery, it is important to recognize the principles of adult education. These principles were the basis for the Training the Trainers Manual: Learning and Teaching of the Royal College of Surgeons of England (RCSE) [3]. In cooperation with the RCSE the manual was transformed into a course by the German Society of Surgeons. Unfortunately this concept was only poorly received by the German Medical Association, which is responsible for the postgraduate medical education in Germany. The program was offered by the Surgical Society as an optional course, but was stopped due to a lack of uptake by residents in preparation for leading roles in surgery. Teach the teacher programs are offered from some medical faculties or university hospitals. The Harvard Surgical Leadership Program [4] for example is a postgraduate certificate program for surgeons seeking to step into and succeed in leadership positions and acquire the skills needed to excel as heads of departments, divisions, projects, and institutions at large. The curriculum highlights modern leadership strategies and executive skills for surgeons, the role of the surgeon as an entrepreneur and innovator and legal principles for surgical leaders.

Within Europe, the working time directive has led to a system of shifts and rotas, which has along with a shorter overall period of training led to reduced time available to surgical trainees in which to learn their craft. In the light of this reduced training time, assessment of surgical competency is becoming even more important and should be a professional priority as well with patient safety and political imperatives in mind. It remains a concern that extended shifts in medical residency programs may adversely affect patient safety but also regarding the effect of current surgical resident duty-hour policies on patient outcomes, resident education, and resident well-being. Allowing program directors flexibility in adjusting duty-hour schedules for trainees did not adversely affect 30-day mortality or several other measured outcomes of patient safety [5]. Compared with standard duty-hour schedules, flexible, less-restrictive duty-hour policies for surgical residents could not be associated with inferior patient outcomes and showed no significant difference in residents' satisfaction with overall well-being and education quality [6].

1. Thomas WEG. Teaching and Assessing Surgical Competence. Ann R Coll Surg Engl. 2006 September; 88(5): 429-432
2. Skills for the new millennium: The CanMeds 2000 Projekt chttp://www.royalcollege.ca/rcsite/canmeds/canmeds-framework-e (11.3.2019)
3. The Royal College of Surgeons of England. Training the Trainers Manual: Learning and Teaching. London: RCSE, 2006.
4. Harvard Surgical Leadership Program https://s3.amazonaws.com/hms-surgery/Harvard+Medical+School+-+Surgical+Leadership+Program.pdf (11.3.2019)
5. Silber JH, Bellini LM, Shea JA et al for the iCOMPARE Research Group. Patient Safety Outcomes under Flexible and Standard Resident Duty-Hour Rules. N Engl J Med 2019; 380:905-914
6. Bilimoria KY, Chung JW, Hedges LV et al. National Cluster-Randomized Trial of Duty-Hour Flexibility in Surgical Training. N Engl J Med 2016; 374:713-727

Surgical competence and its assessment is one of the most hotly debated topics engaging the profession. The present paper gives an overview and describes how leadership is understood and imparted to candidates for the position of a leader in Polish surgery. After the reorganization of the education system, the paper is based on three years of experience of the leading institution for intensive recruitment and training of certified educationists at the Medical University of Lublin, the workplace of the author of this paper.

In the ongoing debate about surgical leadership it seems a worthwhile contribution. The paper it is recommended for publication in the present form after a minor revision:

Page 6: Please explain the term and meaning of "National Consultant" and the Concept of SurgExcellence

AR: The role of Polish National Consultant was explained in P.5-6 including the description of the SurgExcellence program P.6

Page 7: Please give a short comment to the status of implementation of the SEFEAST project „Safety and interdisciplinary surgical care"

AR:The implementation information has been incorporated into the manuscript

Editorial request:
The relevant information must be given in the Author Statement included in your manuscript before the Reference section as follows:

Author Statement
Research funding: Authors state no funding involved.
Conflict of interest: Authors state no conflict of interest.

AR:Information has been added

Reviewer 1: Matthias Rothmund

Apr 01, 2019

**Reviewer Recommendation Term:**	
**Overall Reviewer Manuscript Rating:**	N/A
**Custom Review Questions**	**Response**
Is the subject area appropriate for you?	5 - High/Yes
Does the title clearly reflect the paper's content?	4
Does the abstract clearly reflect the paper's content?	4
Do the keywords clearly reflect the paper's content?	4
Does the introduction present the problem clearly?	5 - High/Yes
Are the results/conclusions justified?	3
How comprehensive and up-to-date is the subject matter presented?	4
How adequate is the data presentation?	N/A
Are units and terminology used correctly?	N/A
Is the number of cases adequate?	N/A
Are the experimental methods/clinical studies adequate?	N/A
Is the length appropriate in relation to the content?	3
Does the reader get new insights from the article?	4
Please rate the practical significance.	4
Please rate the accuracy of methods.	N/A
Please rate the statistical evaluation and quality control.	N/A
Please rate the appropriateness of the figures and tables.	N/A
Please rate the appropriateness of the references.	3
Please evaluate the writing style and use of language.	4
Please judge the overall scientific quality of the manuscript.	4
Are you willing to review the revision of this manuscript?	No: This manuscript needs no further revision. It was revised according to the reviewers comments.

Comments to Authors:

I think this manuscript was well revised and can be published without further changes.

Reviewer 2: anonymous

Apr 01, 2019

**Reviewer Recommendation Term:**	Accept
**Overall Reviewer Manuscript Rating:**	70

Custom Review Questions	Response
Is the subject area appropriate for you?	5 - High/Yes
Does the title clearly reflect the paper's content?	5 - High/Yes
Does the abstract clearly reflect the paper's content?	5 - High/Yes
Do the keywords clearly reflect the paper's content?	5 - High/Yes
Does the introduction present the problem clearly?	5 - High/Yes
Are the results/conclusions justified?	5 - High/Yes
How comprehensive and up-to-date is the subject matter presented?	5 - High/Yes
How adequate is the data presentation?	5 - High/Yes
Are units and terminology used correctly?	N/A
Is the number of cases adequate?	N/A
Are the experimental methods/clinical studies adequate?	N/A
Is the length appropriate in relation to the content?	5 - High/Yes
Does the reader get new insights from the article?	5 - High/Yes
Please rate the practical significance.	5 - High/Yes
Please rate the accuracy of methods.	N/A
Please rate the statistical evaluation and quality control.	N/A
Please rate the appropriateness of the figures and tables.	N/A
Please rate the appropriateness of the references.	5 - High/Yes
Please evaluate the writing style and use of language.	5 - High/Yes
Please judge the overall scientific quality of the manuscript.	5 - High/Yes
Are you willing to review the revision of this manuscript?	Yes

Comments to Authors:
Thank you very much for your prompt revision with the addiotinal explanation of the issues mentioned in my comment. It leads to a better understanding of the special structures in your country
